# The distribution of SNPs in human gene regulatory regions

**DOI:** 10.1186/1471-2164-6-140

**Published:** 2005-10-06

**Authors:** Yongjian Guo, D Curtis Jamison

**Affiliations:** 1 School of Computational Sciences, George Mason University, Manassas, VA 20110 USA; 2 Virginia Bioinformatics Institute, Bioinformatics Facility I (0477), Virginia Tech, Blacksburg, VA 24060 USA

## Abstract

**Background:**

As a result of high-throughput genotyping methods, millions of human genetic variants have been reported in recent years. To efficiently identify those with significant biological functions, a practical strategy is to concentrate on variants located in important sequence regions such as gene regulatory regions.

**Results:**

Analysis of the most common type of variant, single nucleotide polymorphisms (SNPs), shows that in gene promoter regions more SNPs occur in close proximity to transcriptional start sites than in regions further upstream, and a disproportionate number of those SNPs represent nucleotide transversions. Additionally, the number of SNPs found in the predicted transcription factor binding sites is higher than in non-binding site sequences.

**Conclusion:**

Current information about transcription factor binding site sequence patterns may not be exhaustive, and SNPs may be actively involved in influencing gene expression by affecting the transcription factor binding sites.

## Background

Genetic variation has been found to be a ubiquitous phenomenon, and forms the genetic basis for species diversity. Currently, there are sequence variant data accumulated for humans [1], mouse [2], soybean [3] and other organisms. With the completion of Human Genome Project [4], the study of genetic variation has become one of the keystones in biomedical research, not only because it affects an individual's anthropometric characteristics but also because it influences risk of disease and response to environmental challenges [5]. The information derived from the study of variation not only deepens our understanding of human genes and evolution, but also brings benefits to the identification and treatment of human genetic diseases.

There are several different types of genetic sequence variants, including single nucleotide polymorphisms (SNPs), small deletion and insertion polymorphisms (indels), micro-satellite markers, and polymorphic insertion elements such as retrotransposons [6]. Because the most common variants are SNPs, the term is often abused as a synonym for genetic sequence variation. However, here we restrict its usage to the formal SNP definition: a single base change at a single position.

As a result of high-throughput genotyping methods, millions of human SNPs have been reported in recent years. To more efficiently study those with significant biological functions, a practical method is to concentrate efforts on SNPs located in genomic regions with important functions. There have been several studies focusing on evaluating how SNPs impact phenotype. For example, Ramensky et al. [7] have applied several rules to predict biological effects amino acid substitutions made by non-synonymous coding SNPs. Clifford et al. [8] have explored non-synonymous coding SNPs effects on protein function through exploring those introducing amino acid alterations in protein motif regions.

Another genome region important to gene function are gene regulatory regions. Through binding of specific transcription factors, gene promoters are directly involved in gene transcription initiation and regulation. Thus sequence variation in gene promoters may alter transcription factor identification and binding, which in turn can influence gene expression and effect biological impacts. For example, it has been found that one allele of the HLA-G gene (-725G), whose products inhibits maternal anti-fetal immune response, is highly associated with increasing risk for miscarriage [9]. One possible explanation is that the SNP falls within the binding site of interferon response factor-1 (IRF-1), affecting IRF-1 binding and down-regulating transcription of the HLA-G gene.

Here we analyze the distribution of SNPs in human gene regulatory regions. Putative transcription factor binding sites in the gene promoters were computationally derived and compared to previously identified SNPs. The results show that SNPs have differential distribution characteristics both in gene regulatory regions and in transcription factor binding sites when compared to the entire genomic SNP population.

## Results

The build 33 release of the genome sequence contains 545 contig sequences mapped across all of the Homo sapiens chromosomes. 19,741 gene sequences were extracted from NCBI RefSeq database, which were linked to contig sequences by 15,803 LocusLink entries. While most loci have only one gene, some have two or more forming the structure of a gene cluster. In addition, sequence version discrepancies between the databases lead to the removal of approximately 3000 genes. The loss of these genes does not create any bias, as the errors in the affected sequence records were randomly distributed across the databases with no discernible pattern. Thus the final sequence data consisted of derived promoter regions for 16,429 genes.

TFD contains 3,749 mammalian transcription factor binding site sequences, with approximately 2,000 of them being found at least once in the derived gene promoter sequences. More than 1,700 binding sites were not found, presumably because they are either non-human, located outside the immediate upstream gene regions, or are binding sites for trans-regulatory elements. The number of predicted binding sites per promoter follows a normal distribution.

### SNP distribution in gene promoters

Over 35,000 SNPs were found in the gene regulatory regions. More than 99.8% of them are two state alleles. The nucleotide substitution rates of promoter SNPs with two alleles are shown in Table 1. For comparison, substitution rates for all SNPs in the dbSNP database are also listed. In both sets of SNPs, transition class SNPs account for approximately two-thirds of the total number. This is not surprising, as the mismatches made by substitution within the same chemical group is thermodynamically more stable [10]. The remaining one-third SNPs are roughly evenly distributed across the four transversion types.

**Table 1:** Nucleotide substitution rate. Nucleotide substitution rates for SNPs in promoter regions (P) and all SNPs genomewide (A) show roughly similar rates for transition and transversion substitution, with a slight increase in CG transversions as would be expected from CG rich regions.

Type	Substitution	Frequency (P)	Frequency (A)
Purine	A ↔ G	31.55%	33.10%
Pyrimidine	C ↔ T	30.99%	33.10%
Purine ↔ Pyrimidine	C ↔ G	12.75%	8.93%
Purine ↔ Pyrimidine	A ↔ C	9.43%	8.77%
Purine ↔ Pyrimidine	G ↔ T	9.33%	8.82%
Purine ↔ Pyrimidine	A ↔ T	5.94%	7.42%

**Figure 1:**
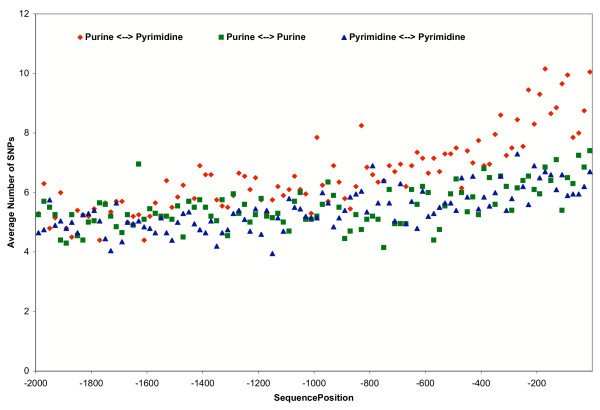
**Distributions of SNPs in different categories in gene promoter regions**. Each symbol represents the average number of SNPs found in a 20 bp bin across approximately fourteen thousand promoter sequences with a full length of 2000. This composite statistic shows the SNP density increases in proximity to the transcriptional start site.

For SNPs in promoter regions, G/C substitution is higher than other nucleotide substitutions in the transversion type, which corresponds to the higher GC content in gene promoters. The distributions of SNPs of different types are plotted in Figure 1, which demonstrates that promoter SNPs are not evenly distributed in the promoter range of [-2000, -1], with more SNPs are observed in the region close to the transcriptional start site. While the numbers of SNPs of all types are all increased in the region near transcriptional start site, the number of SNPs of transversion type shows the largest increase.

### Transcription factor binding site redundancy

Data redundancy of transcription factor binding site motifs was evaluated by comparing binding site sequence similarity, which was divided into three categories: exact coverage, partial coverage and single nucleotide difference. The results of these three categories are shown in Table 2. There are 310 pairs of binding sites with exact sequence coverage such that the sequences can be represented using more generalized patterns. There are 1,114 pairs with partial coverage properties that can be regarded as a subsequence of other binding sites. Finally, 833 pairs are found with sequences that differ only at a single nucleotide position. Potential data redundancy of the binding site sequences makes it uninformative to study individual binding sites in the partial coverage and single nucleotide difference categories, so these motif pairs were merged into single motifs.

**Table 2:** Transcription factor binding site sequence similarity. Examples of the three classes of degeneracy in binding site sequence motifs. For motif pairs with exact coverage, the less restrictive motifs were used. Motif pairs in the partial coverage and single difference categories were merged into a composite motif.

Category	Pair Number	Binding Site Examples	
		
		Sequence	Site Name
Exact Coverage	310	YYCCGCCC CCCCGCCC	EARLY-SEQ1 (Sp1)-TK.1
Partial Coverage	1,114	TGGNNNNNNGCCA YTGGCANNNTGCCAR	NFI_CS3 TGGCA_RS
Single Difference	833	TKCTGATTGTYTMM TKCTGATTGGYTMM	E-alpha_Y_box NF-Y_CS

### Transcription factor binding sites distribution in gene promoters

**Figure 2:**
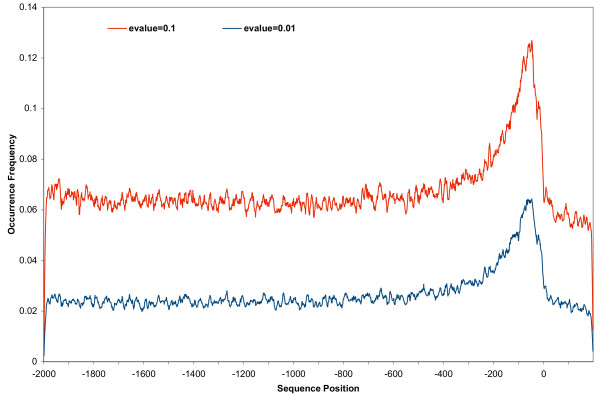
**Distribution of transcription factor binding sites in gene promoter regions**. The x-axis is the position index of gene promoter sequence, and the y-axis is the occurrence frequency of the binding sites across all promoters. Only genes with full length promoter sequences were included in calculation (approximately 14,000 sequences). A portion of the downstream sequence relating to transcriptional start site was also included. The position with index of 0 specifies the transcriptional start sites.

The distribution of binding sites in the gene promoter regions is not uniform, as shown in Figure 2. In the range of -2000 to -400 upstream the frequency is fairly constant. However the frequency increases in the regions closer to the transcriptional start site, reaching two to three times higher in the -100 to -50 range than that in the further sequence regions. This suggests most transcription factor binding sites occur within 250 bp of the initiation site. Beyond the transcriptional start site the frequency of binding sites decreases dramatically, with the frequency value dropping below that of the entire upstream sequence.

Changing the expectation value from 0.1 to 0.01 causes the overall transcription factor binding site frequency to become smaller. The decrease is uniform across the entire sequence range of -2000 to 200, as fewer binding site sequence patterns fulfilling the match criteria are detected. This uniformity suggests there is no issue of selective sensitivity.

**Figure 3:**
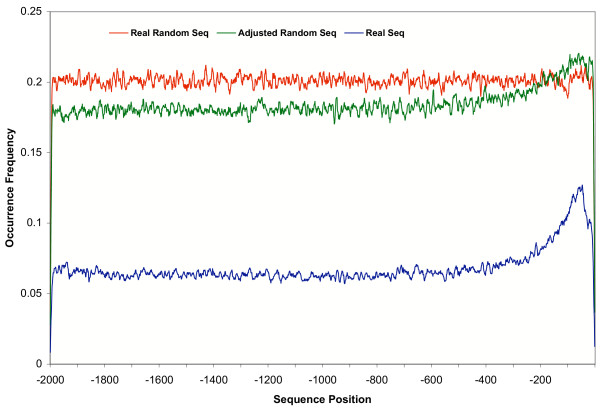
**Comparison of transcription factor binding site distributions in random sequence datasets and real promoter sequence dataset**. Each curve represents the occurrence frequency of predicted binding sites in different data sets of comparable size. "Real Random Seq" is a data set of completely random sequences in which the emission probabilities of A, C, G and T were equal and uniform across the entire 2 kb. "Adjusted Random Seq" is a data set of random sequences generated with the adjusted emission probabilities of A, C, G and T according to that in the corresponding position at the real promoter sequence. "Real Seq" is the real promoter sequence dataset. An expectation value of 0.1 was used for detecting transcription factor binding sites in these datasets.

Transcription factor binding sites in random sequence datasets simulating promoter sequence in the range of -2000 to -1 were predicted using an expect value 0.1, and the results are shown in Figure 3. There is a measurable difference in the occurrence of transcription factor binding sites in the real promoter sequence dataset and the randomly generated sequence dataset. Three-fold more binding sites are detected in the random sequences than in the real promoter sequences, suggesting a non-random nucleotide distribution in real promoter sequences.

The patterns of transcription factor binding site occurrence in the two random sequence datasets are different. For completely random sequences, the occurrence appears to be flat across the whole range. However, in the frequency-based random sequences an increased appearance is observed in the sequence range close to transcriptional start sites similar to the pattern in the real promoter dataset. As the only difference between these two random datasets is the nucleotide emission probabilities at each sequence position, it suggests that transcription factor binding sites are biased by sequence composition. As GC content is enriched in the regions close to transcriptional start site [11], the fact that more transcription factor binding sites are observed in the promoter region near transcriptional start sites indicates that the binding site sequences are GC skewed.

### SNP distribution in transcription factor binding sites

For 34,858 promoter SNPs mapped to 13,723 promoter regions, 2,078 (5.9%) of them are located in the predicted transcription factor binding sites, and 1,969 (5.6%) of them have alleles introducing new binding sites. The overlap of these two SNP sets has 243 members, indicating that their alleles may have different effects on transcription factor binding.

The nucleotide frequency of SNPs falling inside and outside transcription factor binding sites is shown in Table 3. The frequency of SNP distribution in the overall gene promoter region is 0.13%. However, the frequency increases to 0.20% (p = 2.2 × 10^-16) ^in the predicted transcription factor binding site regions.

**Table 3:** Promoter SNPs distribution on predicted transcription factor binding sites. Frequency of SNPs appearing in promoter regions and in putative transcription factor binding sites. The frequency of SNPs is significantly higher in putative binding sites than in the surrounding sequence (p = 2.2 × 10^-16^).

Categories	SNPs	Nucleotides	Frequency
Inside a binding site	3,804	1,890,176	0.20%
Outside the binding site	31,054	25,555,824	0.12%
Total	34,858	27,446,000	0.13%

A set of 293 experimentally derived transcription factor binding sites (associated with 85 genes) drawn from TRANSFAC were linked to predicted transcription factors used for this study. Of these, 13 contained SNPs, as shown in Table 4. The total number of nucleotides organizing the 293 TF binding sites is 5131. With 13 SNPs, the frequency of SNPs in the TF binding site sequence is 0.25%, a value not significantly different from the frequency of promoter SNPs in the putative TF binding sites (p-value = 0.348).

**Table 4:** Promoter SNPs distribution on experimental transcription factor binding sites. Genes which have SNPs in experimentally determined transcription factor binding sites. The frequency is not significantly different from the frequency of promoter SNPs in the putative TF binding sites (p-value = 0.348).

Gene Accession ID	SNP Accession ID	SNP Position	TF Binding Site Start	TF Binding Site End	Binding Site Name	TRANSFAC Binding Site ID
NM_000384	rs9282608	-149	-155	-134	ApoB-site [25]	R03692
NM_000384	rs13306199	-82	-86	-61	AF-1-k-apoB [26]	R01612
NM_005252	rs4645852	-84	-85	-78	c-fos.5 [27]	R00470
NM_002467	rs4645940	-1827	-1834	-1820	c-myc-PUR [28]	R04301
NM_002467	rs13250910	-322	-326	-310	NHE [29]	R01804
NM_002690	rs2307158	-83	-93	-75	Sp1-human-beta-polymerase [30]	R00287
NM_002690	rs2307155	-55	-72	-51	CREB-beta-polymerase [31]	R00288
NM_000805	rs9889551	-100	-102	-95	gastrin-negative-element [32]	R02031
NM_000176	rs10482604	-708	-713	-691	GRFE [33]	R03301
NM_000600	rs13447445	-64	-80	-64	NF-kappaB-IL-6 [34]	R01634
NM_000208	rs1864010	-494	-511	-486	Sp1-insulin_receptor [35]	R03287
NM_002123	rs2854271	-106	-117	-99	HLA-DQB1-Xbox [36]	R03695
NM_001063	rs8177185	-617	-623	-599	transferrin-undefined-site [37]	R01453

## Discussion

Gene promoters for this study were derived using the human genome contig sequences and the RefSeq gene sequences, which are synthesis products of NCBI GenBank [12] records and other information sources. One concern is whether the gene sequences have reliable transcriptional start sites. The traditional experimental methods for identifying transcriptional start sites, such as S1 nuclease mapping [13], primer extension [14] and 5' RACE [15] are technically difficult and not always reliable. Consequently, the Eukaryotic Promoter Database [16], which collects the data from literatures, has only several hundred data entries.

In contrast, the Database of Transcriptional Start Sites [17] uses the full length cDNA library created by oligo-capping [18] to capture the longest mRNA transcript, and has accumulated more than 400,000 sequence entries. These cDNA sequences have been compared with the gene sequences in the RefSeq database to find the target genes and used to study the difference of the transcriptional start sites in the two datasets. According to DBTSS, for 8397 full length cDNA sequences whose corresponding RefSeq gene have been identified and compared, more than 85% of them have transcriptional start site differences equal to or less than 50 nucleotides. Thus the RefSeq gene sequences are relatively reliable in defining the transcriptional start sites.

Although more than 85% of RefSeq gene sequences have almost exact transcriptional start sites, it is possible that some of the derived upstream sequences may not be real gene promoters. The impact of false promoters on the observations is minimal however, as the statistics are calculated using composite statistics. The use of composite statistics also reduces potential bias introduced by highly studied genes, where there might be a greater number of SNPs simply due to a greater number of sequenced individuals, and bias due to the greater interest in the 200 bp immediately upstream of the transcription start site. The normal distribution of binding sites per promoter also argues against any bias due to frequently studied genes, because if there were such study bias we would expect a bimodal distribution with the frequently studied genes having more SNPs forming one mode, and the less studied genes with fewer SNPs forming the second.

Transcription factor binding site prediction algorithms tend to over-predict sites. However, correlation of experimentally determined sites with the predicted sites showed no significant deviation in the number of SNPs falling within confirmed or predicted transcription factor binding sites. The nucleotide frequency of SNPs in experimental transcription factor binding site sequence is 0.25%, which is comparable with the 0.20% nucleotide frequency of promoter SNPs in the putative TF binding sites, and significantly higher than the 0.13% overall nucleotide frequency in the promoter sequences used in this study. Thus we are reasonably certain that the observations reflect a real phenomenon.

When transcription factor binding sites are mapped on gene promoter regions, the anchoring transcriptional start site forms a separating point as shown in Figure 2. Upstream of the transcriptional start site, occurrence frequency of binding sites goes up, whereas the frequency is reduced downstream of the start site. It is clear that SNP distribution in gene promoter sequences is not even. SNPs occur more often in regions close to transcriptional start site than further regions, as is shown Figure 1. This conforms to other observations that the SNP distribution in human chromosomes is not uniform [19].

In general, SNP frequency is directly related to the evolutionary pressure on the target genome regions. For example, more SNPs are accumulated in repetitive sequence, introns and pseudogenes, as the evolution pressure in these regions is relatively low compared to functional gene sequence regions. Given this hypothesis, it is difficult to explain why more SNPs are observed in the sequence region close to transcriptional start site, as the region is important to the initiation of gene transcription, and sequence alteration has potentials to influence gene expression.

One possible explanation is that higher SNP frequency is related to important functions of the regions close to transcription start sites. The accumulation of SNPs in the human genome is like a snapshot of human evolutionary history in which genes, especially those with specific functions, are under continuous natural selection pressure and alteration by mutation, genetic drift and gene flow. As a result, the expression pattern of a gene may be changed. While some genes become totally inactive, others experience expression level alteration. It is possible that SNPs occurring in gene promoter regions play an important role in such scenario, so that the higher frequency of SNPs close to transcriptional start site is related to subtle alteration of gene expression which results in population diversity.

Nucleotide changes caused by SNPs can be classified as transitions and transversions. Generally, transitions are more conservative, because the substituted nucleotides belong to the same chemical group, purine or pyrimidine. In contrast, SNPs resulting in transversions involve nucleotide substitution across purine and pyrimidine chemical groups. The different chemical structures normally limit the occurrence of SNPs of transversion type, however such SNPs have increased frequency in regions close to transcriptional start site as shown in Figure 1. Comparison of nucleotide substitution rates for SNPs in gene promoter regions versus the entire genome also demonstrates that transversion SNPs are increased in promoter regions. It is notable that SNPs with C/G substitution are significantly increased, suggesting that SNPs with unfavorable structure substitution are indeed selected for in the gene promoter regions close to transcriptional start site.

One explanation for the increased SNPs of transversion type may lie in the gene transcription mechanism. During gene transcription, the DNA double helix needs to be opened so that one strand can be used as the template for producing mRNA. In the DNA double helix structure, there are two hydrogen bonds between A and T base pairs and three hydrogen bonds between G and C base pairs, thus opening a G-C base pair requires more energy than an A-T base pair opening does. GC-rich regions often require formation of protein-DNA complexes to facilitate helix opening. Involvement of proteins allows tighter control mechanisms on gene transcription so that gene expression can more accurately respond to environmental conditions. An increased frequency of SNPs with C/G substitutions may be evolutionarily selected for to maintain higher GC content and allowing changes of transcription regulation based on sequence alteration.

It is curious that there are more SNPs in binding site regions than in normal sequence regions. This is contradictory to the hypothesis that transcription factor binding sites are highly conserved because they are important to gene transcription regulation. It is possible that the observations are an artifact and the identified putative transcription factor binding sites are not real binding sites for human genes. As the binding site sequence patterns used are in the mammalian category of the TFD database, some transcription factor binding sites may belong to organisms other than human. However, the false positive binding sites would be simply normal sequence regions in gene promoter which should have the lower genomic SNP frequency, biasing the frequency downward rather than upward.

A second potential artefact is quality control in the transcription factor binding site data used. While data redundancy may exist as shown in Table 2, it is possible that some binding site sequence patterns are not exhaustive. Thus, identified SNPs in the binding sites may have no influence on the binding site integrity and therefore will not affect transcription factor binding and gene expression. This hypothesis cannot be ruled out computationally and will require experimental results to confirm if a SNP found in a transcription factor binding site can exert a real effect on gene expression. If not, the sequence pattern of the binding site needs to be expanded. However, the sequence patterns used for prediction represent consensus sequences drawn from experimental data, and are probably relatively stable and exhaustive.

A third explanation for increased observation of SNPs in the binding sites is that SNPs are involved in altering gene expression during evolution through affecting the binding site sequences. Effects of SNPs in the binding site is likely not simple. While some binding sequence changes made by SNPs may totally interrupt gene expression, others may only influence the level of expression. Considering that gene transcription is a complex process involving many transcription factors, a single position change may not influence all of them. In evolution, the requirement for adjusting certain gene expression level to certain environmental factors forms a natural selection on gene regulation, on which SNPs occurring in transcription factor binding site have direct effect. Therefore, the fact that more SNPs are in the binding sites may demonstrate diverse requirements for different gene expression under different condition.

## Conclusion

A biological explanation for increased observation of SNPs in the binding sites may be that SNPs are involved in altering gene expression during evolution by affecting the binding site sequences. The effects of SNPs in the binding site is likely not simple. While some binding sequence alterations made by SNPs may totally interrupt gene expression, others may only influence the level of expression. Considering that gene transcription is a complex process involving many transcription factors, a single position change may not influence all of them.

In evolution, the requirement for adjusting certain gene expression level to certain environmental factors forms a natural selection on gene regulation, on which SNPs occurring in transcription factor binding site have direct effect. Therefore, the fact that more SNPs are in the transcription factor binding sites may demonstrate diverse requirements for different gene expression under different condition. Thus, promoter SNPs may be an active factor in natural selection on gene transcription.

## Methods

**Figure 4:**
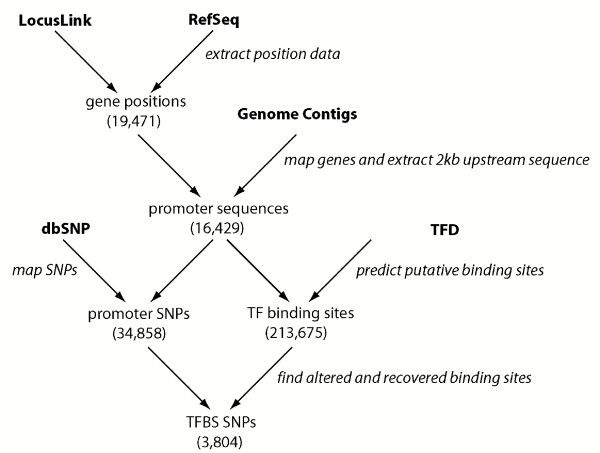
**Flow chart of analysis steps used to generate data set**. A high-level view of the methods used to generate the data sets used in this study. Canonical data sources are shown in bold, and general steps are shown in italics. Parenthetical numbers indicate the number of unique data elements in the data set.

Data from four different sources (LocusLink, Genome, TFD, and dbSNP) were used to generate the promoter SNP data set. At each step, a number of steps to clean and normalize the data were undertaken. Figure 4 shows the general steps and data sources used in our investigation.

### Gene promoter identification

Gene promoter sequences were calculated using human genome contig sequences (build 33) from NCBI and gene location information from LocusLink. For single gene loci, the gene was mapped onto its target contig using the scope value, reverse complementing the contig sequence as necessary. The 2 kb sequence immediately upstream of the transcriptional start site was then extracted and used as the promoter sequence. For genes located closer than 2 kb to the start of contig sequence, the length of the extracted upstream sequence was limited to the available sequence. Loci having more than one gene suggest that genes are co-transcribed, or gene products resulting from alternative splicing. Therefore, the start site of the first gene in the locus was used to derive the upstream sequence, which was used as the promoter sequence for all of the genes in the locus. The few conflicts from data discrepancies across different NCBI databases were resolved by forcing agreement on sequence version in the data extraction pipeline.

### SNP distribution in gene promoters

Promoter SNPs were mapped using the SNP location information from dbSNP [6]. Non-unique and loosely mapped SNPs accounted for only 5.5% for the total number of SNPs and were excluded. SNPs were then classified according to the substitution type as either transitions, which includes nucleotide substitution within the purine (A/G) or pyrimidine (C/T) group, or as transversions, which represents substitution across the groups. The general distribution pattern of SNPs was then computed.

### Data redundancy of transcription factor binding sites

Transcription factor binding site sequences were acquired from the Transcription Factor Database (TFD) [20]. The database contains more than three thousand mammalian binding site sequence entries encoded using the nomenclature of International Union of Pure and Applied Chemistry (IUPAC) [21]. Inspection of the sequence data revealed that some sequences differ at only one or two nucleotides, and some are subsequences of other sequences. To evaluate possible data redundancy, the binding site sequences were mutually compared and the sequence pairs were grouped into three categories: exact coverage (a complete match), partial coverage (one is a subsequence of the other) and single nucleotide difference (differing at an internal nucleotide). Binding sites in the partial coverage and single nucleotide difference categories were merged.

### Transcription factor binding site identification and distribution in gene promoters

Transcription factor binding sites were predicted using a suffix tree algorithm [22,23]. The IUPAC codes were fully expanded and placed into a suffix tree, which allows the exact promoter sequence string to be searched against the binding site motifs while preserving the degeneracy of the binding sites. The probability of the binding site motif appearing by chance was computed using an expectation value given by the equation , where *k *is the number of possible nucleotides at position *i*.

The distribution of binding sites in gene promoters was examined in order to identify the relation between occurrence of binding sites and location in gene promoter regions. Calculations were performed only upon the full length (2 K bp) promoters sequence. A 200 bp portion of the gene subsequence just downstream of the transcriptional start site was also included with the promoter sequences. Expectation values of 0.1 and 0.01 were used as the control for binding site significance.

To evaluate the chance appearance of transcription factor binding sites, we computed their occurrences in two HMM-generated random datasets that simulate the promoter sequence range of [-2000, -1]. The first dataset was created by assigning equal an emission probability to all four nucleotides at each position, while for the second dataset the emission probability of nucleotide was determined according to the observed nucleotide probabilities from the real promoter dataset. Both simulated data sets contained the same number of sequences as the real promoter set.

### SNP distribution in putative binding sites

Integrity of transcription factor binding sites was analyzed by comparing the SNP distributions within and outside the putative transcription binding sites. The transcription factor binding site data set contained both the binding sites directly identified from the canonical sequence, and the binding sites "recovered" by substituting previously identified SNP into the sequence.

### SNP distribution in experimentally derived binding sites

Experimentally derived transcription factor binding sites were retrieved from TRANSFAC database 6.0 [24], the eukaryotic transcription factors database. To evaluate whether SNPs occur in the experimentally derived TF binding sites, we linked the predicted TF binding sites with the binding sites in TRANSFAC using RefSeq literature references. The initial linkage was then confirmed using binding site sequence patterns, site locations and the order of the binding sites in gene promoter.

## Authors' contributions

YG conceived of the study and carried out the coding and statistical analysis. DCJ participated in its design and coordination, and helped draft the manuscript.
